# Two sympatric new species of woodlizards (Hoplocercinae, ***Enyalioides***) from Cordillera Azul National Park in northeastern Peru

**DOI:** 10.3897/zookeys.277.3594

**Published:** 2013-03-15

**Authors:** Pablo J. Venegas, Omar Torres-Carvajal, Vilma Duran, Kevin de Queiroz

**Affiliations:** 1 División de Herpetología-Centro de Ornitología y Biodiversidad (CORBIDI), Santa Rita N°105 Of. 202, Urb. Huertos de San Antonio, Surco, Lima-Perú; 2 Escuela de Biología, Pontificia Universidad Católica del Ecuador, Avenida 12 de Octubre y Roca, Apartado 17-01-2184, Quito-Ecuador; 3 Department of Vertebrate Zoology, National Museum of Natural History, Smithsonian Institution, MRC 162, Washington, DC 20560, USA

**Keywords:** Cordillera Azul, *Enyalioides*, Hoplocercinae, new species, Peru, ystematics, Cordillera Azul, *Enyalioides*, especie nueva, Hoplocercinae, Perú, sistemática

## Abstract

We report the discovery of two sympatric new species of *Enyalioides* from a montane rainforest of the Río Huallaga basin in northeastern Peru. Among other characters, the first new species is distinguishable from other *Enyalioides* by the combination of the following characters: strongly keeled ventral scales, more than 37 longitudinal rows of dorsals in a transverse line between the dorsolateral crests at midbody, low vertebral crest on the neck with vertebrals on neck similar in size to those between hind limbs, projecting scales on body or limbs absent, 96 mm maximum SVL in both sexes, and caudals increasing in size posteriorly within each autotomic segment. The second new species differs from other species of *Enyalioides* in having strongly keeled ventral scales, scales posterior to the superciliaries forming a longitudinal row of strongly projecting scales across the lateral edge of the skull roof in adults of both sexes, 31 or fewer longitudinal rows of strongly keeled dorsals in a transverse line between the dorsolateral crests at midbody, vertebrals on neck more than five times the size of vertebrals between hind limbs in adult males, projecting scales on body or limbs absent, and caudals increasing in size posteriorly within each autotomic segment. We also present an updated molecular phylogenetic tree of hoplocercines including new samples of *Enyalioides rudolfarndti*, *Enyalioides rubrigularis*, both species described in this paper, as well as an updated identification key for species of Hoplocercinae.

## Abstract (es)

Reportamos dos nuevas especies simpátricas de *Enyalioides* provenientes de los bosques lluviosos montanos de la cuenca del Río Huallaga. La primera especie nueva se distingue de las demás especies de *Enyalioides* por la combinación de los siguientes caracteres: ventrales fuertemente quilladas, mas de 37 filas longitudinales de dorsales (contadas en línea transversal entre la crestas dorsolaterales a la mitad del cuerpo), cresta dorsal muy baja con las vertebrales del cuello de una talla similar a las vertebrales que se encuentran entre las piernas, falta de escamas proyectadas sobre el cuerpo o miembros, talla pequeña (con una longitud máxima de hocico a cloaca de 96 mm en ambos sexos) y caudales que aumentan en tamaño posteriormente en cada segmento autotómico. La segunda especie nueva se diferencia del resto de especies de *Enyalioides* por tener ventrales fuertemente quilladas, escamas posteriores a las superciliares formando una fila longitudinal de escamas fuertemente proyectadas a lo largo del borde lateral del techo del cráneo (en ambos sexos), 31 o menos filas longitudinales de dorsales fuertemente quilladas (contadas en línea transversal entre las crestas dorsolaterales a la mitad del cuerpo), vertebrales sobre el cuello más de cinco veces la talla de las vertebrales que se encuentran entre las piernas en machos adultos, ausencia de escamas proyectas sobre el cuerpo o miembros y caudales que aumentan en tamaño posteriormente en cada segmento autotómico. Además, presentamos un árbol filogenético molecular de hoplocercinos actualizado que incluye nuevas muestras de *Enyalioides rudolfarndti*, *Enyalioides rubrigularis*, las nuevas especies reportadas aquí, así como una clave de identificación actualizada para las especies de Hoplocercinae.

## Introduction

Woodlizards (*Enyalioides*) are represented by ten currently recognized extant species that occur between 0–2000 m on both sides of the Andes from Panama to Bolivia (Torres-Carvajal et al. 2011). Eight species, the largest number for a single country, occur in Ecuador followed by Peru (7), Colombia (5), Brazil (2), Panama (1), and Bolivia (1) (Torres-Carvajal et al. 2011; Venegas et al. 2011). Although *Enyalioides* has been regarded as a group of low species diversity, recent fieldwork combined with taxonomic analyses has revealed a previous underestimation of species diversity. Three of the ten species, *Enyalioides touzeti*, *Enyalioides rubrigularis*, and *Enyalioides rudolfarndti*, have been described since 2008 (Torres-Carvajal et al. 2008, 2009; Venegas et al. 2011). These species were discovered in recent expeditions to poorly explored areas on both sides of the Andes in Ecuador and Peru, suggesting that more species might be awaiting discovery in other unexplored areas close to the Andes.

The 1.3 million ha Cordillera Azul National Park (CAZNP) is the third largest National Park in Peru and protects the largest extent of montane rainforest in the country. This national park is located between the Huallaga and Ucayali rivers, and includes some of the least explored forests of four Regions of Peru (i.e., Huánuco, Loreto, San Martín, and Ucayali). Rodríguez et al. (2002) reported 58 species of amphibians and 26 species of reptiles from the basins of the rivers Pisqui and Pauya in CAZNP (Loreto). In recent surveys at CAZNP in the San Martín Region we collected specimens of *Enyalioides* that are inferred to represent two new species, based on morphological and molecular evidence, and are reported on in this paper. This discovery increases the number of species of woodlizards known to occur in Peru to nine, making it the country with the highest known species diversity in this clade.

## Materials and methods

The type series of the new species reported on in this paper were deposited in the herpetological collection of the Centro de Ornitología y Biodiversidad (CORBIDI) in Lima, Peru. Specimens of other *Enyalioides* species from CORBIDI, the Museo de Historia Natural San Marcos (MUSM) in Lima, Peru, and the Museo de Zoología, Pontificia Universidad Católica del Ecuador (QCAZ) in Quito, Ecuador were examined for comparative purposes and are listed in Appendix 1. Snout-vent length(SVL) and tail length (TL) measurements were made with a ruler and recorded to the nearest millimeter. Allother measurements were made with digital calipersand recorded to the nearest 0.1 mm. Sex was determinedby noting the presence/absence of hemipenes. We followedthe terminology of Avila-Pires (1995) and Torres-Carvajal et al. (2011) for scutellational characters and measurements.

### Phylogenetic analyses

Torres-Carvajal and de Queiroz (2009) sampled two nuclear genes (BDNF, RAG1) and a continuous fragment of mitochondrial DNA (mtDNA) that extends from the protein-coding gene ND1 (subunit one of NADH dehydrogenase) through the genes encoding tRNA^ILE^, tRNA^GLN^, tRNA^MET^, ND2 (subunit two of NADH dehydrogenase), tRNA^TRP^, tRNA^ALA^, tRNA^ASN^, the origin of light-strand replication, tRNA^CYS^, tRNA^TYR^, to the protein-coding gene COI (subunit I of cytochrome c oxidase) to examine phylogenetic relationships among hoplocercine species. Following similar laboratory protocols, we sequenced the mtDNA fragment for five specimens of the new species reported herein (CORBIDI 6772, 8825–28), as well as three specimens of *Enyalioides rudolfarndti* (CORBIDI 7209–10, 7212) and one of *Enyalioides rubrigularis* (QCAZ 8454). GenBank accession numbers are KC588838–KC588846, respectively. We added these new sequences to the mtDNA dataset of Torres-Carvajal and de Queiroz (2009), and followed their alignment and model selection protocols. Phylogenetic relationships were assessed under a Bayesian approach using MrBayes 3.2.0 (Ronquist and Huelsenbeck 2003) after partitioning the data (tRNAs, 1^st^, 2^nd^, and 3^rd^ codon positions of protein coding genes). To reduce the chance of converging on a local optimum, four runs were performed. Each consisted of five million generations and four Markov chains with default heating values. Trees were sampled every 1000 generations resulting in 5000 saved trees per analysis. Stationarity was confirmed by plotting the –ln *L* per generation in the program Tracer 1.2 (Rambaut and Drummond 2003). Additionally, the standard deviation of the partition frequencies and the potential scale reduction factor (Gelman and Rubin 1992) were used as convergence diagnostics for the posterior probabilities of bipartitions and branch lengths, respectively. Adequacy of mixing was assessed by examining the acceptance rates for the parameters in MrBayes and independence of samples was assessed by examining the effective sample sizes (ESS) in Tracer. After analyzing convergence, mixing, and sampling, the first 500 trees in the sample were discarded as “burn-in" from each run. We then confirmed that the four analyses reached stationarity at a similar likelihood score and that the topologies were similar, and used the resultant 18,000 trees to calculate posterior probabilities (PP) for each bipartition in a maximum clade credibility tree in TreeAnnotator 1.6.1 (Rambaut and Drummond 2010).

## Results

### Enyalioides azulae sp. n.

urn:lsid:zoobank.org:act:FADE520D-B1C5-4C5A-A54D-6E8A682E1E29

http://species-id.net/wiki/Enyalioides_azulae

**Figure 1.**
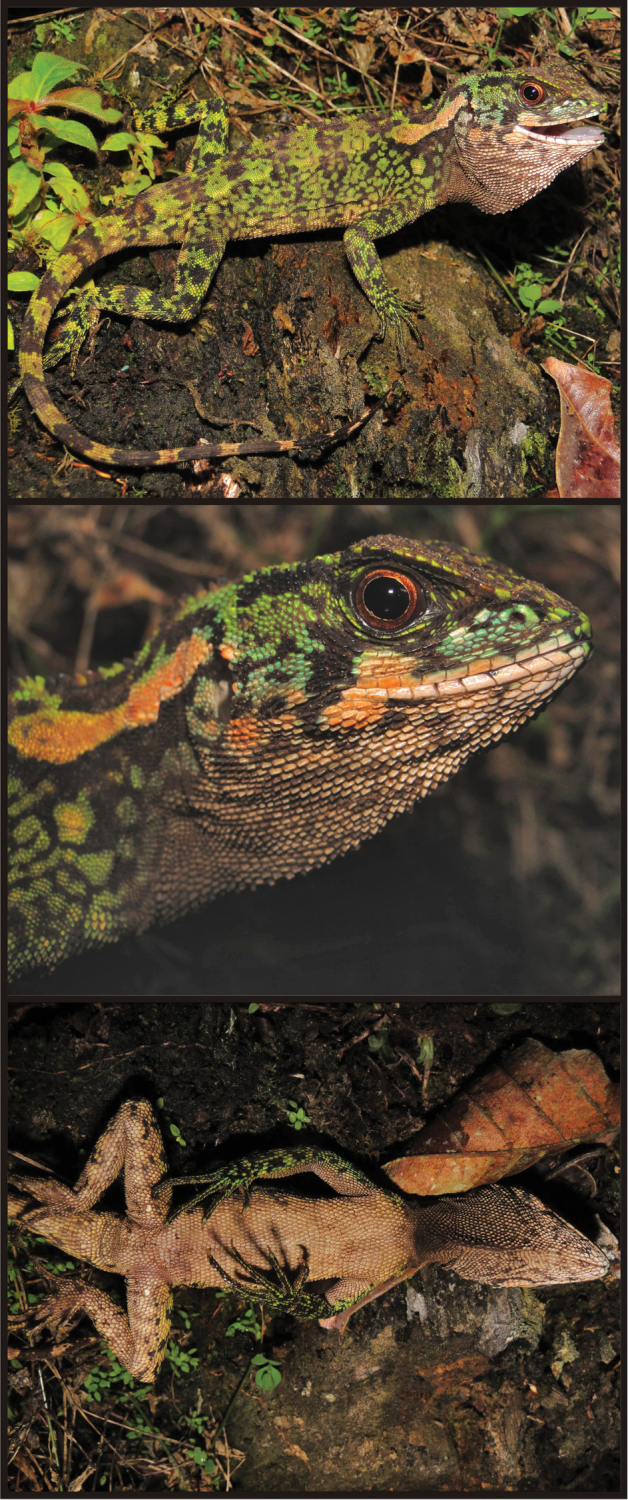
Holotype of *Enyalioides azulae* sp. n. (CORBIDI 06772, adult male, SVL = 96 mm). Top: lateral view; middle: close-up of head; bottom: ventral view. Photographs by P.J. Venegas.

Figs 1 [Fig F2] [Fig F3] –4

#### Holotype.

CORBIDI 06772 (Fig. 1), an adult male from Chambirillo close to Checkpoint 16 of the CAZNP (07°04'8.9"S, 76°00'51.2"W, 1122 m), Provincia de Picota, Región San Martín, Perú, collected on 1 May 2010 by P. J. Venegas.

#### Paratypes.

CORBIDI 8825, 8826, adult females collected on 30 October 2010 by P. J. Venegas; CORBIDI 08786, 08790, 08791, adult male, juvenile female, and juvenile male, respectively, collected on 21 January 2011 by P. J. Venegas and V. Duran; CORBIDI 09213, 09214, juvenile male and female, respectively, collected on 8 May 2011 by P. J. Venegas and V. Duran. All paratypes are from the type locality.

#### Diagnosis.

**Figure 2. F2:**
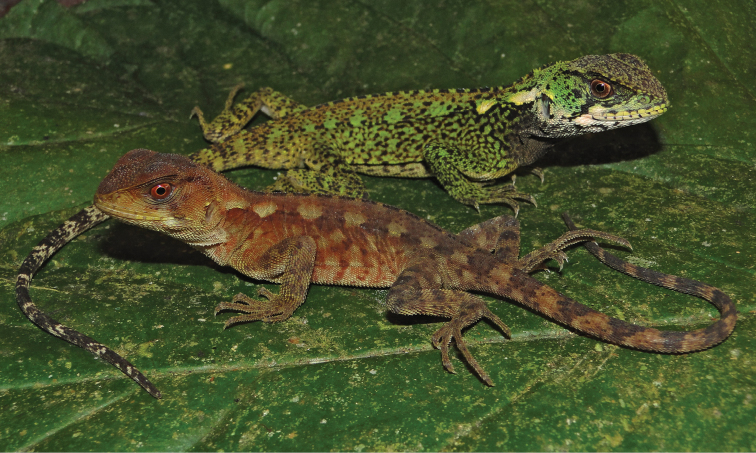
Male (top, CORBIDI 09213) and female (bottom, CORBIDI 09214) of *Enyalioides azulae* sp. n. Photograph by P.J. Venegas.

*Enyalioides azulae* can be distinguished from other species of *Enyalioides*, except *Enyalioides microlepis* and *Enyalioides cofanorum*, by the combination of the following characters: (1) strongly keeled ventral scales; (2) more than 37 longitudinal rows of dorsals in a transverse line between the dorsolateral crests at midbody; and (3) absence of superciliary flaps projecting over each orbit (present only in *Enyalioides palpebralis*). *Enyalioides azulae* differs from *Enyalioides cofanorum* and *Enyalioides microlepis* in having more gulars (45–57, mean = 51.13 ± 4.05, versus 34–41, mean = 36.13 ± 2.00 in *Enyalioides cofanorum* and 34–49, mean = 37.88 ± 3.44 in *Enyalioides microlepis*), a smaller body size (maximum SVL = 96 mm in both males and females, versus 107 mm in males and 109 mm in females of *Enyalioides cofanorum*, and 127 mm in males and 116 mm in females of *Enyalioides microlepis*), a lower vertebral crest on the neck, a narrower snout in dorsal view, and in lacking blue on the gular region in males. Additionally, *Enyalioides azulae* has a marked sexual dichromatism, with males having greenish and females brownish background coloration (Fig. 2), whereas the other two species have brownish background coloration in both sexes. *Enyalioides azulae* further differs from *Enyalioides cofanorum* in lacking scattered enlarged scales on the dorsum, well-developed dorsolateral crests between the hind limbs, and a dark gular patch in females.

#### Description of holotype.

Male (Fig. 1); SVL = 96 mm; TL = 140 mm; maximum head width = 21.28 mm; head length = 26.35 mm; head height = 17.95 mm; dorsal head scales uni- or multicarinate, those on parietal region projected dorsally; parietal eye present; scales immediately posterior to superciliares conical and as dorsally projected as adjacent parietals and temporals; temporal scales small, granular and multicarinate; one enlarged pretympanic scale; 14 superciliares; six canthals; five postrostrals; 11 (left or right) supralabials counted to a point below middle of eye; rostral (2.57 × 1.16 mm) about twice as wide as adjacent supralabials; two longitudinal rows of lorilabials between suboculars and supralabials at level of middle of eye, 3–4 longitudinal rows of lorilabials anterior to this point; loreal region broken into small, multicarinate, and juxtaposed scales; nasal at level of supralabials III–IV; 10 (left or right) infralabials counted to a point right below middle of eye; mental (2.51 × 1.53 mm) wider and longer than adjacent infralabials; two postmentals; gulars ventrally projected; gular fold complete midventrally, extending dorsally and posteriorly to form antehumeral fold; neck with several oblique folds and a dorsolateral row of enlarged scales.

Vertebral crest not strongly projected, with vertebrals on neck similar in size to those between hind limbs; crest bifurcates posteriorly and extends onto tail less than ¼ its length; body flanks between fore and hind limbs without folds; irregular dorsolateral row of 1–2 keeled, enlarged scales (i.e., approximately twice as large as adjacent scales); dorsal scales between dorsolateral scale rows and vertebral crest small, keeled and subimbricate towards vertebral crest, granular towards dorsolateral scale rows; scales on flanks similar in size to lateralmost dorsal scales; ventral scales subimbricate, keeled, subrectangular, with a posterolateral mucron; ventrals more than twice the length of dorsals.

Limb scales keeled and imbricate dorsally and ventrally; scales on dorsal and posterior aspects of thighs keeled and imbricate, with most scales less than half the size of those on anterior and ventral aspects; 19 subdigitals on manual digit IV; 26 subdigitals on pedal digit IV; one femoral pore on each side; tail laterally compressed and gradually decreasing in relative height towards tip; caudal scales strongly keeled and imbricate, moderately increasing in size posteriorly on lateral and dorsal aspects of each autotomic segment; ventral caudals larger than dorsal caudals, with individual vertebral segments three scales long ventrally and six scales long dorsally.

**Color in life of holotype** (Fig. 1). Dorsal surface of head dark brown with light green flecks; lateral surface of head green with lorilabial and pretympanic regions turquoise and a black narrow supratemporal stripe; a black oblique stripe extending from eye to commisure of the mouth; an orange cream oblique stripe on suboculars, posterior labials and adjacent gulars; labials cream; rostral and mental light green; wide, cream longitudinal stripe extending from above tympanum to scapular region; gular region dirty cream with dark spots and flecks; dark brown patch on medial aspect of gular fold; dorsal background green with diffuse, transverse dark brown bars on body, limbs, and tail; flanks covered with dark brown reticulations; ventral surface of body, limbs and tail tan with diffuse darker brown spots on thighs; iris reddish copper with a fine golden ring around the pupil.

#### Intraspecific variation.

**Figure 3. F3:**
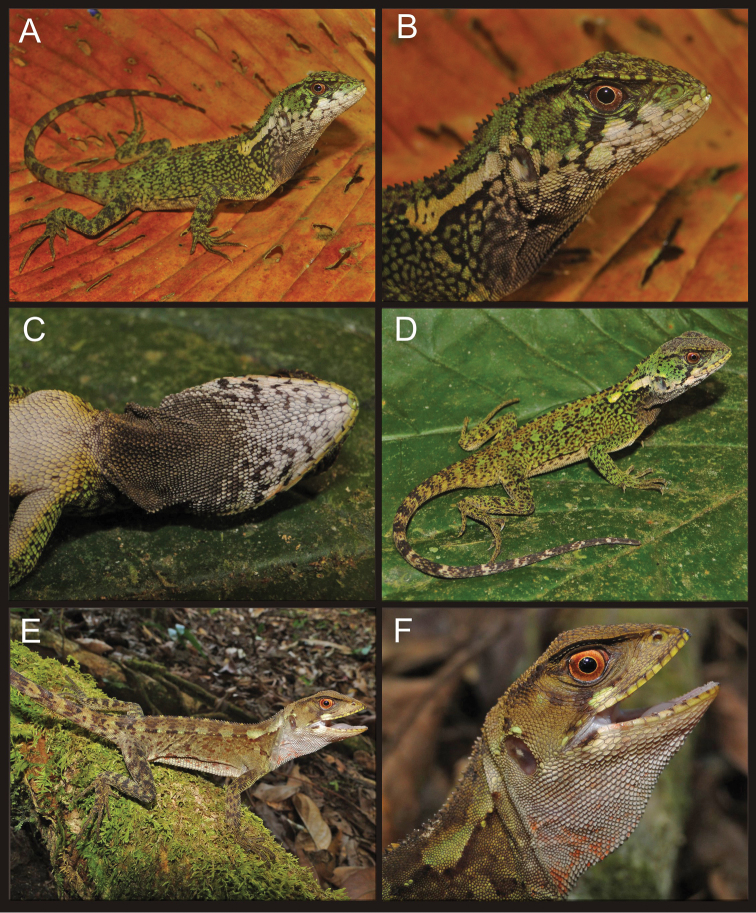
Adult male (**A, B, C** CORBIDI 08786), juvenile male (**D** CORBIDI 09213), and adult female (**E, F** CORBIDI 08826) of *Enyalioides azulae*. Photographs by P.J. Venegas.

Meristic and morphometric characters of *Enyalioides azulae* are summarized in Table 1. Male paratypes (CORBIDI 08786, 09213) are very similar in coloration to the holotype (Fig. 3A–D). The dark patch on the gular region of adult males is also present in juvenile male specimens.

**Figure 4.**
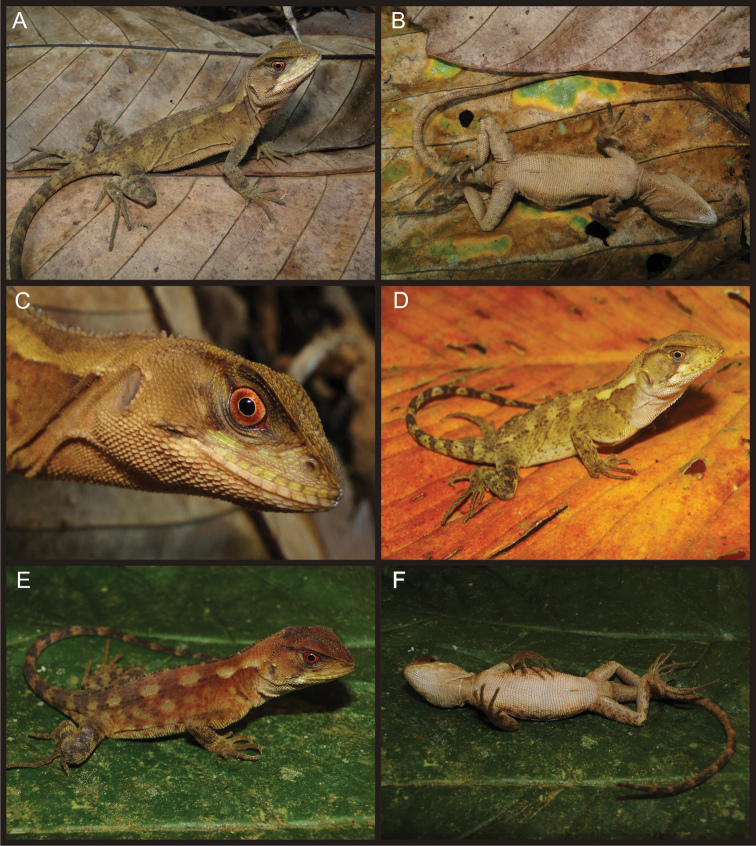
Adult (**A, B, C** CORBIDI 08825) and juvenile (**D** CORBIDI 08791 **E, F** CORBIDI 09214) females of *Enyalioides azulae*. Photographs by P.J. Venegas.

Adult (CORBIDI 08825–08826) and juvenile (CORBIDI 08791, 09214) females share similar color patterns (Figs. 3E–F, 4): head brown with a narrow dark brown supratemporal stripe; broad subocular dark stripe extending from eye to commisure of mouth, with a parallel, conspicuous white or cream stripe immediately anterior to it; pale, wide longitudinal stripe extending from tympanum to scapular region; gular region pale brown without dark markings, or white with faint reddish brown reticulation as in specimen CORBIDI 08826; dorsal background light brown, with a greenish tone in CORBIDI 08826 (Fig. 3E–F) and coppery tone in CORBIDI 09214 (Fig. 4E); transverse dark brown bars on dorsal aspect of body, limbs, and tail; ventral surface of body, limbs and tail light brown (CORBIDI 08825; Fig. 4B) or white (CORBIDI 08826; Fig. 4F); iris reddish brown.

Although this species seems to have a marked sexual dichromatism in background colors (green in males, brown in females, see Fig. 2), one male specimen (the holotype) exhibited metachromatism consisting of dark brown tones being replaced by green tones.

#### Distribution and natural history.

**Figure 5.**
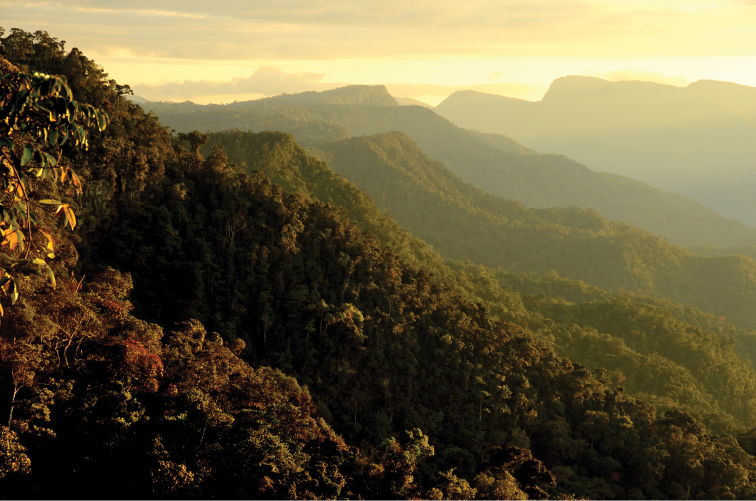
Habitat at the type locality of *Enyalioides azulae* sp. n. and *Enyalioides binzayedi* sp. n. The photo shows the montane rainforest on the top of the mountain ridge that forms the boundary between Region de San Martin and Region de Loreto. Photograph by A. Del Campo.

**Figure 6.**
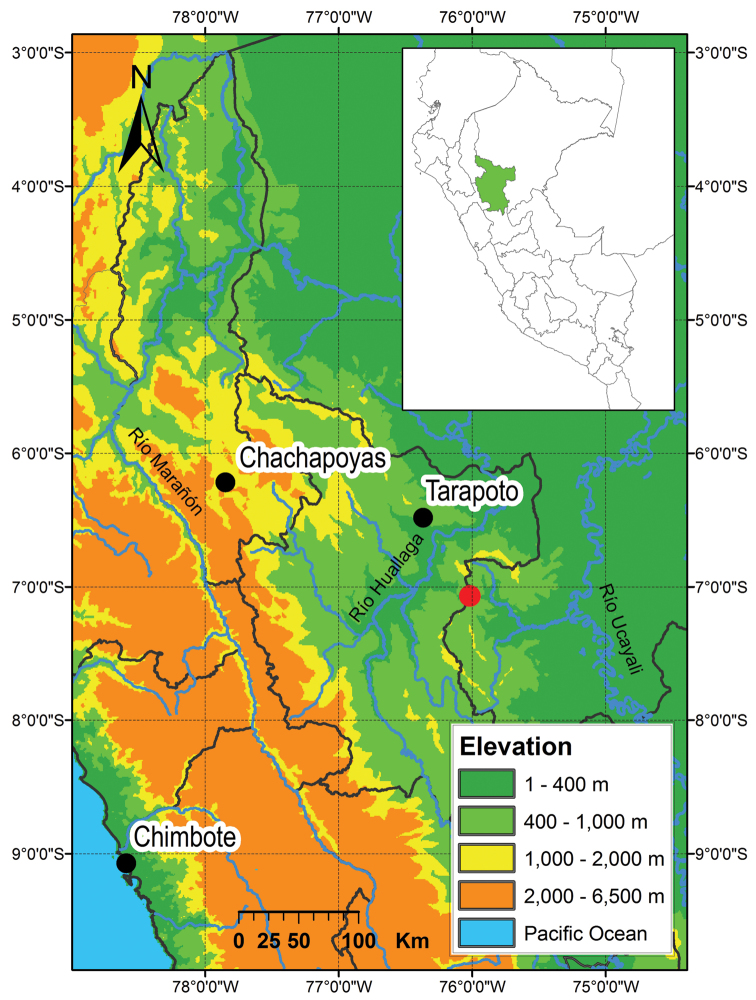
Distribution of *Enyalioides azulae* sp. n. and *Enyalioides binzayedi* sp. n. in Peru. The red circle indicates the type (and only currently known) locality of both species.

*Enyalioides azulae* is known only from its type locality in the montane rainforest of the Río Huallaga basin (Fig. 5) in northeastern Peru at an elevation of 1100 m. This locality lies within the CAZNP, on a mountain ridge between the Región San Martín and Región Loreto (Fig. 6). Seven of the eight individuals of *Enyalioides azulae* reported in this paper were collected at night sleeping on low vertical stems of bushes 15–80 cm above the ground. One adult male (the holotype) was collected during the day on a narrow trail after a rain; when approached, it fled and hid under a fallen log. This species is found in sympatry and possibly syntopy with *Enyalioides binzayedi* sp. n. (see below) and *Enyalioides laticeps*. The smallest individuals (CORBIDI 08790–08791, SVL = 61 and 62 mm, respectively) were collected in January. Other species of squamate reptiles collected at the same locality include *Alopoglossus angulatus*, *Anolis fuscoauratus*, *Anolis transversalis*, *Cercosaura manicata*, *Potamites ecpleopus*, *Potamites strangulatus*, *Potamites* sp., *Chironius fuscus*, *Dipsas indica*, *Imantodes cenchoa*, *Imantodes lentiferus*, *Micrurus obscurus*, *Oxyrhopus petola*, and *Xenopholis scalaris*.

#### Etymology.

The specific epithet is a noun derived from the Spanish word “azul" (blue) in the genitive case; it refers to the Cordillera Azul, the mountain range after which the National Park where this species was discovered is named. Although the word “azul" in “Cordillera Azul" is an adjective, and the Spanish noun “azul" is masculine, we are here treating “azulae" as a feminine noun that is an abbreviation for “Cordillera Azul" and is therefore to be interpreted as meaning “of the [Cordillera] Azul."

### Enyalioides binzayedi sp. n.

urn:lsid:zoobank.org:act:01F81E7A-E9CF-42DA-88F9-6EB2A4EE3EFC

http://species-id.net/wiki/Enyalioides_binzayedi

Figs 7 [Fig F8] –9

#### Holotype.

**Figure 7.**
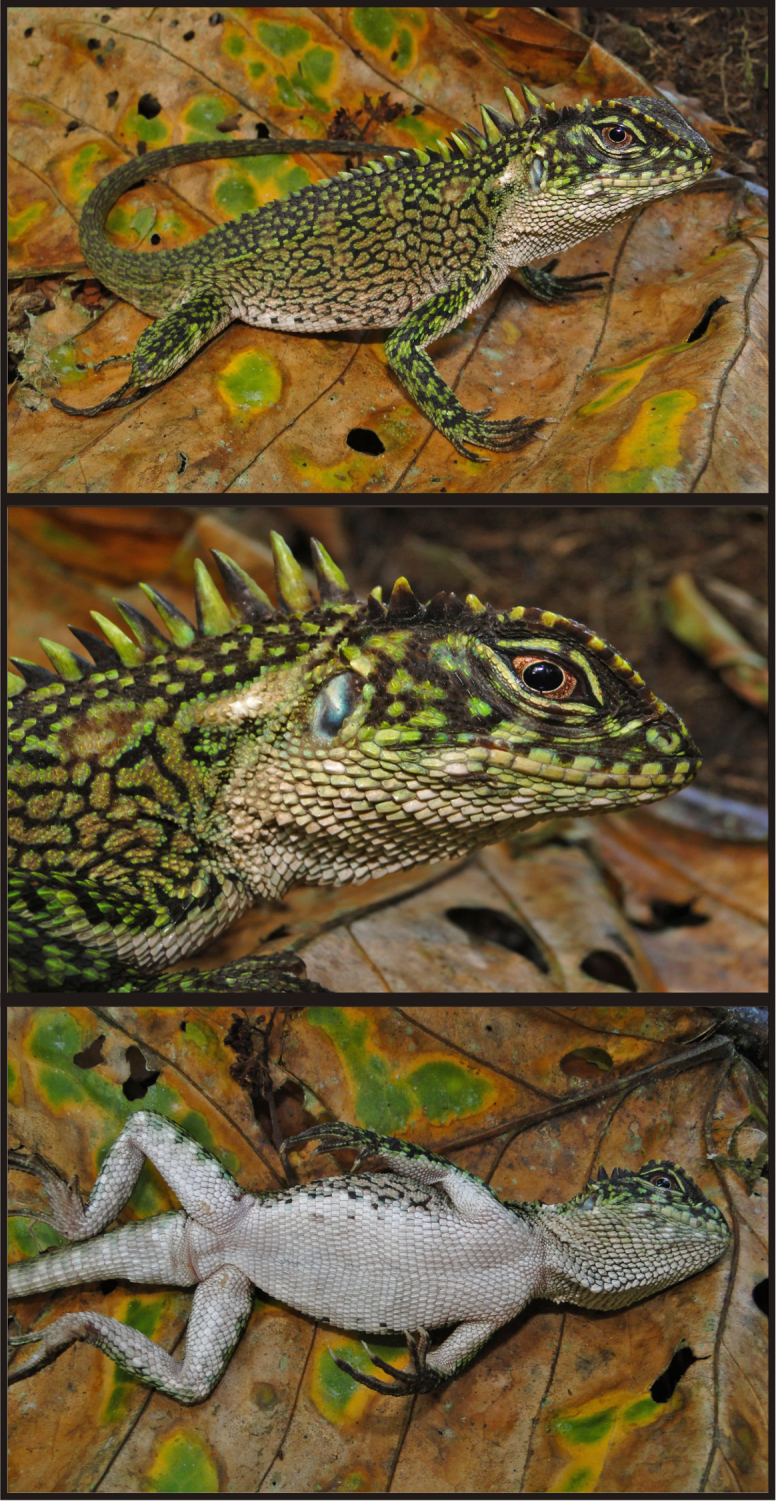
Holotype of *Enyalioides binzayedi* sp. n. (CORBIDI 08828, adult male, SVL = 118 mm). Top: lateral view; middle: close-up of head; bottom: ventral view. Photographs by P.J. Venegas.

CORBIDI 08828 (Fig. 7), an adult male from Chambirillo close to the Checkpoint 16 of the CAZNP (07°04'8.9"S, 76°00'51.2"W, 1122 m), Provincia de Picota, Región San Martín, Perú, collected on 30 October 2010 by P. J. Venegas.

#### Paratypes.

CORBIDI 08827, an adult female collected on 2 November 2010 by P. J. Venegas; CORBIDI 08786, 08787, 08788, 08789, adult females collected on 21 January 2011 by P. J. Venegas and V. Duran; CORBIDI 09215, 09216, a juvenile male and adult female, respectively, collected on 6 May 2011 by P. J. Venegas and V. Duran. All paratypes are from the same locality as the holotype.

#### Diagnosis.

*Enyalioides binzayedi* can be distinguished from other species of *Enyalioides* by the combination of the following characters: (1) scales posterior to the superciliaries forming a longitudinal row of strongly projecting scales across the lateral edge of the skull roof in adults of both sexes; (2) 31 or fewer longitudinal rows of strongly keeled dorsals in a transverse line between the dorsolateral crests at midbody; (3) ventral scales strongly keeled; (4) caudals increase in size posteriorly within each autotomic segment; (5) projecting scales on body or limbs absent; (6) vertebrals on neck more than five times the size of vertebrals between hind limbs in adult males.

A longitudinal row of strongly projecting scales along the lateral edge of the skull posterior and continuous with the superciliaries is also present in *Enyalioides oshaughnessyi*, which occurs west of the Andes in Ecuador and Colombia and differs from *Enyalioides binzayedi* in having smooth or slightly keeled dorsals. Species of *Enyalioides* occurring east of the Andes that share strongly keeled ventrals with *Enyalioides binzayedi* are *Enyalioides azulae*, *Enyalioides cofanorum*, *Enyalioides microlepis*, *Enyalioides palpebralis*,and *Enyalioides rudolfarndti*. All of these species either lack strongly projecting scales along the lateral edge of the skull roof (although they are slightly projecting in *Enyalioides rudolfarndti*) or have such scales but with a gap separating them from the superciliaries (*Enyalioides palpebralis*). *Enyalioides azulae*, *Enyalioides cofanorum* and *Enyalioides microlepis* differ further from *Enyalioides binzayedi* (character states in parentheses) in having more than 33 dorsal scales in a transverse line between the dorsolateral crests at midbody (31 or fewer), a low vertebral crest (high, with vertebrals on neck more than four times the size of vertebrals between hind limbs in both sexes), and a black gular patch (absent). The new species can be also distinguished from *Enyalioides palpebralis* by lacking both a superciliary triangular flap that projects posterolaterally over each eye and a small gap in the vertebral crest in the neck region, and by having femoral pores. From *Enyalioides rudolfarndti* (character states in parentheses), *Enyalioides binzayedi* also differs in having a prominent medial keel on each dorsal scale (medial keel weak or absent), dorsals nearly homogeneous in size (dorsals heterogeneous in size), and in lacking a round orange blotch in the antehumeral region (orange blotch present in adult males).

#### Description of holotype.

Male (Fig. 7); SVL = 118 mm; TL = 180 mm; maximum head width = 25.14 mm; head length = 30.46 mm; head height = 23.70 mm; dorsal head scales uni- or multicarinate, those in parietal region strongly projected dorsally; parietal eye present; scales immediately posterior to superciliares conical and dorsolaterally projected, forming longitudinal row of seven scales that extends posteriorly over supratemporal region, with fifth anteriormost scale more than twice the size of other scales in row; temporal scales small, multicarinate, juxtaposed; two large, projected conical temporal scales dorsal to tympanum, the dorsal one in contact with the supratemporal crest, and the ventral one in contact with an enlarged pretympanic scale; 14 superciliares; four canthals; three postrostrals; 12 (left or right) supralabials counted to a point right below middle of eye; rostral (2.27 × 1.27 mm) slightly wider than adjacent supralabials; single longitudinal row of lorilabials between suboculars and supralabials at level of middle of eye, two longitudinal rows of lorilabials immediately anterior to this point; loreal region broken into small, multicarinate, and juxtaposed scales; nasal at level of supralabials III–IV; 11 (left) or 10 (right) infralabials counted to a point right below middle of eye, respectively; mental (2.47 × 1.89 mm) twice as wide and high as adjacent infralabials; postmentals three; gulars ventrally projected, those immediately anterior to gular fold keeled, mucronate, and imbricate; gular fold complete midventrally, extending dorsally and posteriorly to form antehumeral fold; neck with several longitudinal and oblique folds, and a dorsolateral row of enlarged scales.

Vertebral crest strongly projected and decreasing in size posteriorly, with vertebrals on neck at least four times higher than those between hind limbs; crest bifurcates posteriorly and extends onto tail less than ¼ its length; body between fore and hind limbs with dorsolateral crests and without folds; dorsal scales heterogeneous in size, prominently keeled, and subimbricate; scales on flanks more homogeneous in size than dorsals and less than half their size; ventral scales imbricate, keeled, subrectangular, and mucronate; ventrals as large as largest dorsals.

Limb scales keeled and imbricate dorsally and ventrally; most scales on dorsal and posterior aspects of thighs homogeneous in size, less than half the size of scales on anterior and ventral aspects; 19 subdigitals on manual digit IV; 24 subdigitals on pedal digit IV; femoral pores on each side two; tail laterally compressed and gradually decreasing in relative height towards tip; caudal scales strongly keeled and imbricate, slightly increasing in size posteriorly on lateral and dorsal aspects of each vertebral segment; ventral caudals larger than dorsal caudals, with individual autotomic segments three scales long ventrally and four scales long dorsally.

**Color in life of holotype** (Fig. 7). Dorsal and lateral surface of head dark brown or black, with scattered light green scales (especially on the dorsal surface) and a dark longitudinal supratemporal stripe; supralabials greenish white intercalated with dark brown, infralabials greenish white; rostral and mental light green; gulars white, with greenish-white margins; skin between gulars dark gray; dorsal background of body, limbs, and tail light green, with a dark brown reticulation; a white blotch posterior to tympanum followed by five diffuse pale brown dorsolateral blotches extending from the neck to the base of the tail; ventral surface of body, limbs, and tail white, with a longitudinal row of 4–5 dark gray squarish marks between flanks and venter; iris coppery with a fine brown reticulation; pupil round with pale green margin.

#### Intraspecific variation.

Meristic and morphometric characters of *Enyalioides binzayedi* are summarized in Table 1. The holotype is the only adult male specimen available; it differs from female and subadult male paratypes in having projecting scales on each side of the vertebral crest on the neck. Additionally, female paratypes CORBIDI 08789 and 09216 are unique in having a double vertebral crest from midbody to pelvic region.

**Figure 8. F8:**
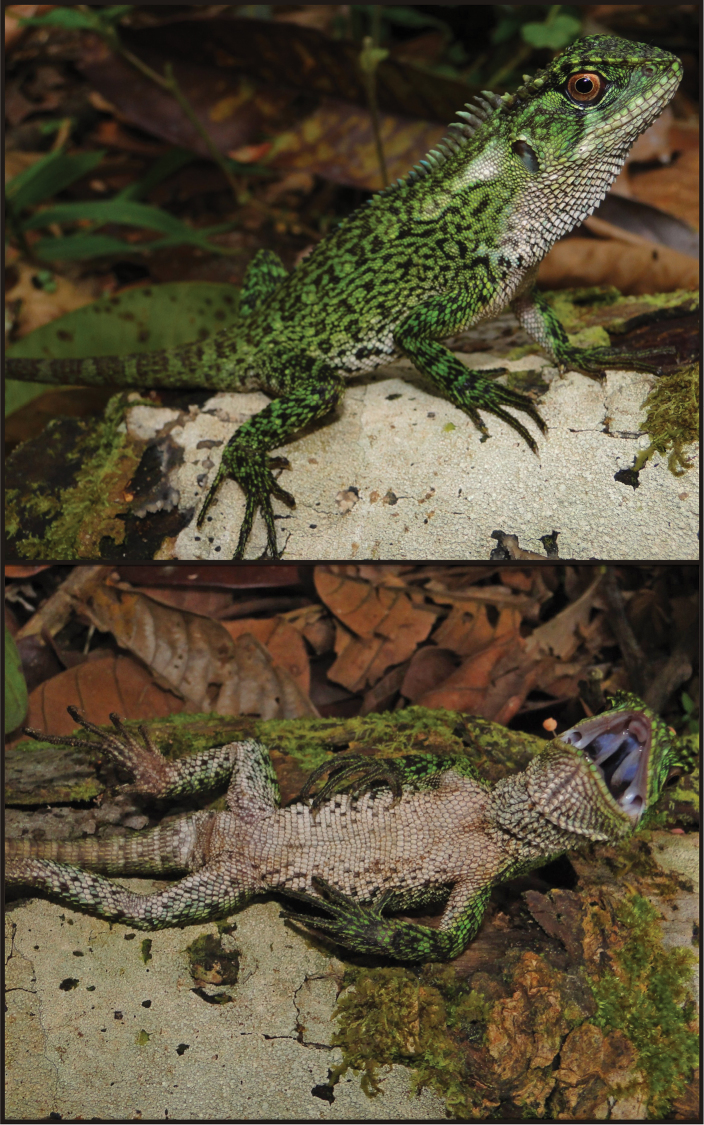
Juvenile male of *Enyalioides binzayedi* sp. n. (CORBIDI 09215). Top: lateral view; bottom: ventral view. Photographs by P.J. Venegas.

**Figure 9.**
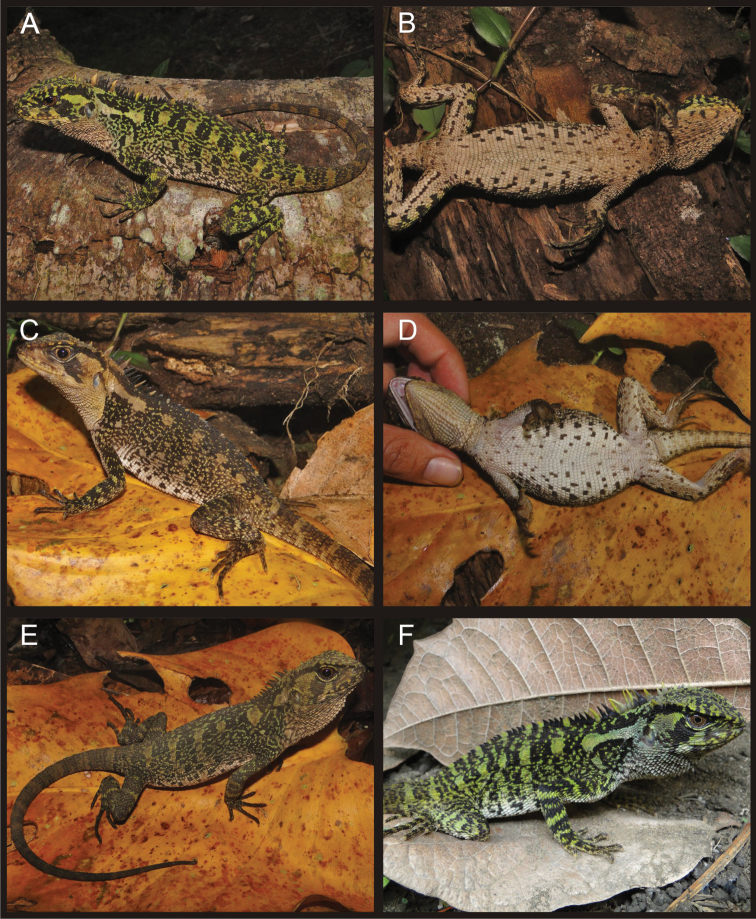
Four adult females of *Enyalioides binzayedi* sp. n. (**A, B** CORBIDI 08787 **C, D** CORBIDI 08788 **E** CORBIDI 08789 **F** CORBIDI 08827). Photographs by P.J. Venegas.

A subadult male specimen (CORBIDI 09215; Fig. 8) differs from the holotype in having scattered black spots on the ventral surface of body. All females differ from the holotype in having dorsal, broad transverse bars arranged longitudinally along the vertebral line, larger dark marks on the ventrolateral surface of body, and well defined postocular and supratemporal stripes. Dorsal background of body, limbs, and tail can be dark greenish brown (CORBIDI 08827 and 08787), as in the holotype, dark green (CORBIDI 08789), or dark brown (CORBIDI 08788) speckled with light green flecks. Females CORBIDI 08787 and 08827 have light dorsolateral blotches intercalating with dark transverse bars, which are well defined dorsolaterally and diffuse laterally (Fig. 9). Female paratypes CORBIDI 08789 and 09216 have a pale blotch behind the tympanum similar to the holotype, whereas CORBIDI 08787, 08827, and 08788 have a larger pale blotch connected to first pale dorsolateral blotch forming a continuous postympanic stripe extending from the tympanum to the scapular region. Ventrally females are white (CORBIDI 08788; Fig. 9D) or tan (CORBIDI 08787, 08789; Fig. 9B) with scattered dark brown spots or flecks. The throat in females is brown or light brown with dark flecks or diffuse reticulations, except one female (CORBIDI 08789), which has an immaculate tan throat.

#### Distribution and natural history.

*Enyalioides binzayedi* is known only from its type locality in the montane rainforest of the Río Huallaga basin (Fig. 6) in northeastern Peru at an elevation of 1080 m. This locality lies within the CAZNP, in a mountain ridge between the Región San Martín and Región Loreto (Fig. 5). All individuals reported here were collected at night sleeping on vertical stems of bushes 30–230 cm above the ground. One female (CORBIDI 08788) collected on 21 January 2011 had two maturing eggs in each oviduct. *Enyalioides binzayedi* occurs in sympatry and possibly syntopy with *Enyalioides azulae* sp. n. (see above) and *Enyalioides laticeps*. Other species of squamate reptiles collected in the same locality include *Alopoglossus angulatus*, *Anolis fuscoauratus*, *Anolis transversalis*, *Cercosaura manicata*, *Potamites ecpleopus*, *Potamites strangulatus*, *Potamites* sp., *Chironius fuscus*, *Dipsas indica*, *Imantodes cenchoa*, *Imantodes lentiferus*, *Micrurus obscurus*, *Oxyrhopus petola*, and *Xenopholis scalaris*.

#### Etymology.

The specific name is a noun in the genitive case and is a patronym honoring Sheikh Mohamed bin Zayed Al Nahyan, Crown Prince of Abu Dhabi and Deputy Supreme Commander of the UAE, who created the Mohamed bin Zayed Species Conservation Fund (MBZSCF) to support species conservation projects around the globe. Field surveys leading to the discovery of the two species reported on in this paper were supported by a grant from the MBZSCF.

##### Phylogenetic relationships

**Figure 10.**
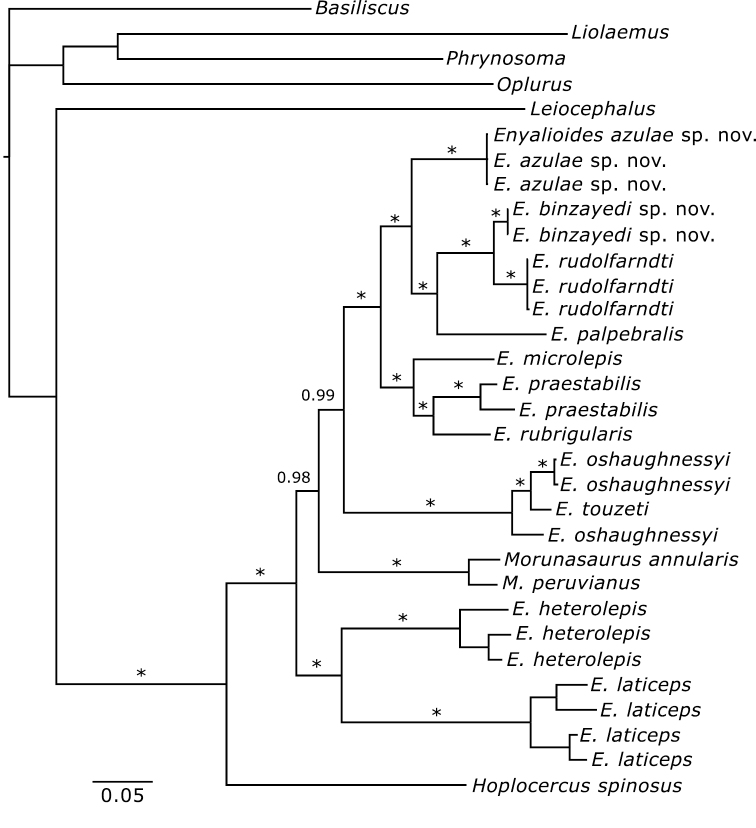
Maximum clade credibility tree of hoplocercine lizards based on a Bayesian analysis of mtDNA sequences. Posterior probabilities are indicated next to branches, with asterisks denoting values > 0.99.

Using a phylogenetic definition (de Queiroz and Gauthier 1990, 1992), Torres-Carvajal and de Queiroz (2009) applied the name *Enyalioides* to the crown clade originating in the most recent common ancestor of *Enyalioides cofanorum* Duellman 1973, *Enyalioides heterolepis* (Bocourt 1874), *Enyalioides laticeps* (Guichenot 1855), *Enyalioides microlepis* (O'Shaughnessy 1881), *Enyalioides oshaughnessyi* (Boulenger 1881), *Enyalioides palpebralis* (Boulenger 1883), *Enyalioides praestabilis* (O'Shaughnessy 1881), and *Enyalioides touzeti* Torres-Carvajal et al. 2008. The phylogenetic tree inferred in this study (Fig. 10) is consistent with Torres-Carvajal and de Queiroz's (2009) phylogenetic hypothesis in that species of *Enyalioides* are split into two primary subclades. One contains *Enyalioides heterolepis* and *Enyalioides laticeps* as sister taxa, and the other includes all remaining species of *Enyalioides*, as well as possibly *Morunasaurus*. *Enyalioides azulae* sp. n. is sister to the clade (*Enyalioides palpebralis*, (*Enyalioides binzayedi* sp. n., *Enyalioides rudolfarndti*)) with strong support (PP = 1.00), whereas *Enyalioides binzayedi* sp. n. is sister to *Enyalioides rudolfarndti* with strong support (PP = 1.00). Both species reported on in this paper, as well as *Enyalioides rudolfarndti*, are strongly supported (PP = 1.00) as monophyletic groups(Fig. 10). Thus, the phylogenetic tree presented here strongly supports both referral of the new species to *Enyalioides* and their status as different species from those recognized previously, except that the divergence between *Enyalioides binzayedi* and *Enyalioides rudolfarndti* is less than that observed within some currently recognized species (*Enyalioides heterolepis* and *Enyalioides laticeps*), which is at least partly attributable to the geographic separation of the samples. Differences in morphology and color patterns presented above provide additional evidence for recognizing *Enyalioides binzayedi* sp. n. and *Enyalioides rudolfarndti* as separate species.

**Table 1.** Summary of counts and measurements (mm) for *Enyalioides azulae* and *Enyalioides binzayedi*. Range (first line) and mean ± standard deviation (second line) are given. Sample size is given in parentheses if different from that in the column heading.

Character	*Enyalioides azulae*; *n* = 8	*Enyalioides binzayedi*; *n* = 7
Vertebrals from occiput to base of tail	62–69 65.88 ± 2.70	40–55 48.00 ± 5.51
Dorsals in transverse row between dorsolateral crests at midbody	37–47 41.63 ± 3.20	22–31 27.57 ± 3.64
Ventrals in transverse row at midbody	27–33 28.75 ± 1.91	26–32 28.14 ± 2.12
Transverse rows of ventrals between fore and hind limb	36–44 40.38 ± 2.45	30–39 35.29 ± 2.81
Gulars	45–57 51.13 ± 4.05	27–31 29.14 ± 1.77
Infralabials	10–13 11.38 ± 1.30	10–14 11.29 ± 1.50
Supralabials	10–14 11.75 ± 1.28	11–15 12.00 ± 1.41
Canthals	4–6 4.63 ± 0.74	4–6 4.43 ± 0.79
Superciliaries	12–18 15.38 ± 2.07	13–14 13.57 ± 0.53
Subdigitals Manual Digit IV	15–22 19.25 ± 1.98	17–22 19.86 ± 1.68
Subdigitals Pedal Digit IV	25–28 26.50 ± 1.07	24–30 27.14 ± 2.48
Femoral pores in males	1 (*n* = 4) —	1–2 (*n* = 2) —
Femoral pores in females	1–2 (*n* = 4) 1.13 ± 0.35	1–3 (*n* = 5) 2.20 ± 0.79
Head length/head width	1.23–1.32 (*n* = 4) 1.26 ± 0.04	1.21–1.41 1.26 ± 0.07
Head width/head height	1.15–1.27 (*n* = 4) 1.20 ± 0.05	1.04–1.16 1.10 ± 0.05
Rostral width/rostral height	1.55–2.22 (*n* = 4) 1.79 ± 0.30	1.51–2.56 1.79 ± 0.36
Mental width/mental height	1.18–1.64 (*n* = 4) 1.41 ± 0.21	1.20–1.63 1.40 ± 0.17
Fore limb length/SVL	0.49–0.53 (*n* = 4) 0.51 ± 0.02	0.47–0.53 0.52 ± 0.02
Hind limb length/SVL	0.75–0.84 (*n* = 4) 0.80 ± 0.04	0.69–0.80 0.75 ± 0.04
Tail length/total length	0.57–0.59 (*n* = 5) 0.58 ± 0.01	0.56–0.60 0.58 ± 0.02
Maximum SVL (mm) males	96 (*n* = 4)	118 (*n* = 2)
Maximum SVL (mm) females	96 (*n* = 4)	122 (*n* = 5)

##### Key to the species of Hoplocercinae

The following key is artificial in the sense that its structure does not necessarily reflect the order of branching in the phylogeny.

**Table:** 

1	Dorsal head scales flat, smooth, juxtaposed; vertebral crest absent or composed of a discontinuous row of enlarged scales that are longer than tall	2
–	Dorsal head scales conical; vertebral crest present, composed by projecting scales that are taller than long	5
2	Tail depressed, short (tail length < snout-vent length), with enlarged spiny scales dorsally and laterally	*Hoplocercus spinosus*
–	Tail nearly round, moderate (tail length > snout-vent length), with rings of enlarged spiny scales	3
3	Vertebral region of trunk without enlarged scales; tail with three scale rows separating the spiny whorls ventrally	*Morunasaurus groi*
–	Some vertebral scales in trunk region enlarged forming a discontinuous longitudinal row; tail with two scale rows separating the spiny whorls ventrally	4
4	Usually two femoral pores on each leg; two postmentals; females without streaks on throat	*Morunasaurus annularis*
–	Femoral pores 3–4 on each leg; usually four postmentals; females with dark streaks on throat	*Morunasaurus peruvianus*
5	Caudal scales homogeneous in size within each autotomic segment	*Enyalioides laticeps*
–	Caudal scales increase in size posteriorly within each autotomic segment	6
6	Laterally projecting superciliary flap present; vertebral crest usually discontinuous (absent on posterior part of neck)	*Enyalioides palpebralis*
–	Laterally projecting superciliary flap absent; vertebral crest continuous	7
7	Scattered, projecting, tetrahedral large scales on dorsum, flanks, and hind limbs present	*Enyalioides heterolepis*
–	Scattered, projecting, tetrahedral large scales on dorsum, flanks, and hind limbs absent	8
8	Ventrals smooth or slightly keeled	9
–	Ventrals conspicuously keeled	10
9	Gulars in males cream or yellow without black margins; usually one femoral pore on each leg	*Enyalioides praestabilis*
–	Gulars in males bright orange or red, with black margins; usually two femoral pores on each leg	*Enyalioides rubrigularis*
10	Dorsals heterogeneous in size, with scattered, tetrahedral, projecting scales (sometimes absent in males or juveniles); dorsolateral crests well developed between hind limbs	*Enyalioides cofanorum*
–	Dorsals homogeneous in size, without projecting scales; dorsolateral crests inconspicuous or absent between hind limbs	11
11	Dorsals smooth or slightly keeled; iris bright red in adult males; dark gular patch, if present, restricted to gular fold in males	*Enyalioides oshaughnessyi*
–	Dorsals conspicuously keeled, iris grey, reddish brown or copper in adult males; dark gular patch, if present, covering gular region in males	12
12	Dorsals in transverse row between dorsolateral crests at midbody 31 or fewer	13
–	Dorsals in transverse row between dorsolateral crests at midbody more than 31	14
13	Scales along the lateral edge of the skull roof strongly projected; dorsal scales homogeneous in size, with prominent median keel; antehumeral orange blotch in adult males absent	*Enyalioides binzayedi*
–	Scales along the lateral edge of the skull roof slightly projected; dorsal scales heterogeneous in size, without prominent median keel; distinct antehumeral orange blotch in adult males	*Enyalioides rudolfarndti*
14	White or cream spot posterior to tympanum usually present; 41–54 (mean = 45.96 ± 3.49) dorsals in transverse row between dorsolateral crests at midbody; gular background in adult maleslight blue	*Enyalioides microlepis*
–	White or cream spot posterior to tympanum absent; 37–47 (means = 41.63 ± 3.20 in *Enyalioides azulae*, 40.50 ± 1.90 in *Enyalioides touzeti*) dorsals in transverse row between dorsolateral crests at midbody; gular background in adult males cream or black	15
15	Vertebral scales in neck region in adult males similar in size as vertebrals in pelvic region; 45–57 (mean = 51.13 ± 4.05) gulars	*Enyalioides azulae*
–	Vertebral scales in neck region in adult males more than twice as high as vertebrals in pelvic region; 42–48 (mean = 44.40 ± 2.22) gulars	*Enyalioides touzeti*

## Supplementary Material

XML Treatment for Enyalioides azulae

XML Treatment for Enyalioides binzayedi

## Appendix I

**Specimens examined**

*Enyalioides cofanorum.-*ECUADOR: *Orellana*:Vía Pompeya-Iro, 66 Km from Pompeya, QCAZ 08035; *Sucumbíos*: La Selva lodge, 0°24'0"S, 76°39'0"W, QCAZ 03953, 03521.

*Enyalioides microlepis.-*Perú: *Loreto*: Pozo Runtusapa, MUSM 22264; Datem del Marañón; Andoas, 3°42'15.6"S, 77°18'46.2"W, 273 m, CORBIDI 01506, 01575; Capahuari Norte, 02°39'51.3"S, 76°30'4.42"W, 270 m, CORBIDI 04804; San Jacinto 02°19'51.0"S, 75°51'49.3", 160 m, CORBIDI 05120.

*Enyalioides palpebralis.-*Perú: *Cusco*: Camisea, 11°35'0"S, 72°57'0"W, 431 m, MUSM 14661; Echarate, 12°50'0"S, 72°39'0"W, 1133 m, MUSM 24663, 26114; 12°34'1.30"S, 73°5'24.9"W, 1300 m, CORBIDI 06042; La Convención 12°11'18.7"S, 73°00'3.31"W, 725 m, CORBIDI 06646, 06752, 06756, 08347; *Loreto*; Requena; Sierra del Divisor, 6°55'7.4"S, 73°50'46"W, 205 m, CORBIDI 02298.

*Enyalioides rudolfarndti.-*Perú: *Pasco*: Huampal 10°11'03"S, 75°34'27"W, 1050 m (Parque Nacional Yanachaga-Chemillen), CORBIDI 07209 (holotype), CORBIDI07210, 07213, 07212 (paratypes).
